# Visual and vestibular reweighting after cyber‐ and space‐sickness

**DOI:** 10.1113/EP092966

**Published:** 2025-06-10

**Authors:** Tess Bonnard, Emilie Doat, Jean‐René Cazalets, Dominique Guehl, Etienne Guillaud

**Affiliations:** ^1^ Université de Bordeaux, CNRS, INCIA, UMR 5287 Bordeaux France; ^2^ Université de Bordeaux, CNRS, IMN, UMR 5293 Bordeaux France

**Keywords:** cyber sickness, multi‐sensorial integration, space sickness, visuo‐vestibular reflexes, weightlessness

## Abstract

Sensory conflicts are widely recognized as the primary drivers of motion sickness (MS), though the underlying integrative processes remain poorly understood. This study investigated sensory reweighting following exposure to two different sensory conflict paradigms. Visual and vestibular reflexes were assessed before and after sensory conflict. In the first paradigm, participants were exposed to a visuo‐vestibular conflict using visually induced illusory motion (vection) in two environments in immersive virtual reality. In the second paradigm, vestibular conflict was induced by gravitational changes in parabolic flight. Semi‐circular canal integration was measured via the vestibulo‐ocular reflex (VOR) suppression task, while visual weight was assessed through optokinetic nystagmus (OKN). Our findings revealed that, following virtual reality exposure, VOR response decreased by 12%, indicating a reduced reliance on vestibular inputs. Conversely, after parabolic flight, OKN performance was diminished by 13%, indicating a diminished weight of visual inputs. These findings suggest that the sensory modality failing to detect the motion was considered less reliable and therefore assigned a reduced contribution during the integration process, regardless of its actual accuracy. Additionally, visual sensitivity was associated with increased susceptibility to cybersickness, whereas vestibular sensitivity seemed to correlate MS severity in parabolic flight. Altogether, our data suggest that the sensitivity of the most stimulated sensory modality during a sensory conflict may predict an individual's susceptibility to MS.

## Abstract (highlights)

- **What is the central question of this study?**
  How does brief exposure to different types of motion sickness influence sensory reweighting?
- **What is the main finding and its importance?**
  Sensory reweighting is elicited through brief and specific exposure to motion sickness, with adaptive responses varying between space‐induced and Earth‐like motion cues. Regardless of their reliability, motionless cues are consistently downweighted. Furthermore, the severity of motion sickness is modulated by individual sensitivity to the activated sensory inputs, which differs across various provocative sensory environments.

## 1 INTRODUCTION

Motion sickness (MS) affects a substantial portion of the population, with symptoms ranging from mild discomfort (e.g. nausea, pallor, cold sweats) to more severe impairments, including cognitive and physical performance deficits (Golding, 2016; Zhang et al., 2016). These symptoms can have critical implications, potentially hindering astronauts' ability to perform highly skilled tasks during the initial days of space travel and after landing without supporting medical staff (Heer & Paloski, 2006), or increasing discomfort and raising safety concerns for autonomous car passengers (Pereira et al., 2024). Current treatments, such as pharmacological solutions (scopolamine for instance), often carry side effects that induce sleepiness and drowsiness, increase imbalance, or decrease visual acuity, which are all incompatible with tasks requiring focus and precision (Bestaven et al., 2016; Weerts et al., 2014). Despite its prevalence, the integrative mechanisms underlying MS remain poorly understood and largely theoretical, with limited identification of effective predictive factors.

The MS symptoms might arise from disruptions in the brain's ability to integrate sensory information about motion and spatial orientation. Sensory signals from multiple modalities converge in cortical and subcortical structures, where they are integrated and weighted based on their consistency with other sensory inputs (Choi et al., 2023; Spence et al., 2004). While the human sensory systems has evolved over millions of years, the rapid emergence of modern technologies – such as cars, airplanes and virtual reality – has introduced novel sensory combinations. These changes have increased the likelihood of sensory conflicts during multisensory integration. These environments often create sensory discrepancies that human brains are typically not exposed to in natural environments. The sensory conflict theory, one of the most widely accepted frameworks, posits that MS arises from contradictory sensory inputs (Bos, 2011; Guedry et al., 1998; Irmak et al., 2023; Reason & Brand, 1975). When sensory systems provide conflicting information about motion or orientation, the central nervous system (CNS) struggles to reconcile these signals, leading to discomfort and other symptoms.

MS can be categorized based on the sensory modalities that trigger it. Broadly, two main types can be distinguished: visuo‐vestibular (inter‐sensory) conflict and vestibulo‐vestibular (intra‐sensory) conflict (Gallagher & Ferrè, 2018; Reason, 1978). Visuo‐vestibular conflict arises from incoherences between visual and vestibular inputs and is commonly experienced during various modes of transportation, such as reading in a moving car or traveling on a windowless boat. It is also prevalent in emerging virtual reality (VR) technologies. In VR, users are immersed in a full‐field artificial visual environment that induces a sensation of vection (self‐motion perception), despite their physical body remaining largely static, seated, or exhibiting restricted movement. This sensory conflict between visual motion perception and the absence of corresponding bodily movement results in cybersickness (Kim et al., 2005), which affects approximately 60–95% of VR users (Caserman et al., 2021; Cobb, 1999). In contrast, vestibulo‐vestibular conflict does not stem from contradictions between different sensory systems but rather from inconsistencies within the vestibular system itself. This phenomenon occurs in environments with altered gravitational conditions, such as spaceflight or parabolic flights (PF), where individuals are suddenly exposed to weightlessness. Humans, having evolved under Earth's gravity, are accustomed to a relatively stable gravitoinertial vector as detected by the otolith organs, with only minor variations. This otolithic input is typically congruent with semicircular canal detection of head rotations, forming the basis of vestibular processing that the brain has adapted to over evolutionary time (Bertolini et al., 2015; Merfeld et al., 2005a, 2005b). However, in highly altered gravitational environments, the otoliths provide unexpected signals, while the semicircular canals continue detecting angular accelerations in the usual manner. This novel sensory combination leads to an otolitho‐canalar conflict, which has been proposed as the origin of space motion sickness (SMS) (Graybiel et al., 1975; Graybiel & Lackner, 1977). SMS affects approximately two‐thirds of astronauts during their initial days in space and one‐third of medicated flyers during parabolic flights (Golding et al., 2017; Lackner & DiZio, 2006). Notably, prolonged exposure to weightlessness leads to otolith tilt‐translation reinterpretation (Parker et al., 1985), a phenomenon in which otolithic stimulation become perceived more as translation than tilt, mirroring the conditions experienced in space. This reinterpretation highlights the importance of adaptative internal models in sensory integration and serves as the foundation for another approach to explaining MS: the Neural Mismatch Theory. This theory suggests that MS arises from discrepancies between current sensory inputs and prior expectations based on past experiences (Reason, 1978). Ultimately, both sensory conflict and neural mismatch theories emphasize the central role of sensory integration in the development of MS.

One theorical framework to explain multisensory integration relies on the convergence of various sensory inputs generated by a single stimulus, using a weighted linear combination of individual perceptual estimates (Ernst & Banks, 2002). It has been proposed that this process serves to reduce perceptual uncertainty about the stimulus (Knill & Pouget, 2004). When one sensory signal is incongruent with the majority of other signals, it introduces greater uncertainty into the integrative process, compromising the accuracy of perception. To address this, the integration mechanism tends to suppress the source of uncertainty, ensuring a more coherent and reliable final perception. The Maximum Likelihood Estimation (MLE) model incorporates these principles, essentially functioning on the idea that ‘the most reliable input will be weighted more heavily than others in the final integration’ (Ernst & Bülthoff, 2004). According to the MLE model, space‐sickness resulting from otolith–canal conflict could diminish the perceived reliability of vestibular input, leading to a downweighting of vestibular cues. Conversely, intense visual stimulation in a stationary participant could compromise the credibility of visual input, reducing the weight assigned to visual motion detection.

Our study aims to investigate whether different sensory conflict paradigms (virtual reality and parabolic flight) elicit distinct sensory reweighting strategies, as reflected in changes of low‐level reflexes (VOR and OKN). Rather than assuming a common effect of motion sickness, we aimed to test whether the source of the sensory conflict determines the direction of multisensory integration. The sensory paradigms were specifically chosen because they reliably induce MS through different mechanisms: visuo‐vestibular mismatch in VR and otolith–canal conflict in PF. By comparing their effects on vestibulo‐ocular reflex (VOR) and optokinetic nystagmus (OKN), we seek to determine whether the brain's reweighting of sensory inputs differs depending on the type of conflict, or whether MS, regardless of its sensory origin, results in common integration strategies. Our protocol involved short exposures (10–20 min) known to provoke MS, enabling us to probe acute changes in reflexive responses immediately post‐conflict. We hypothesized that the sensory modality most affected by the conflict would be down‐weighted post‐exposure, reflected in decreased sensitivity to its associated motion cues (e.g., reduced OKN after VR; reduced VOR after PF). Additionally, we examined whether individual MS severity was related to pre‐existing sensory sensitivity, offering insight into predictive factors for MS vulnerability.

## 2 METHODS

**FIGURE 1:**
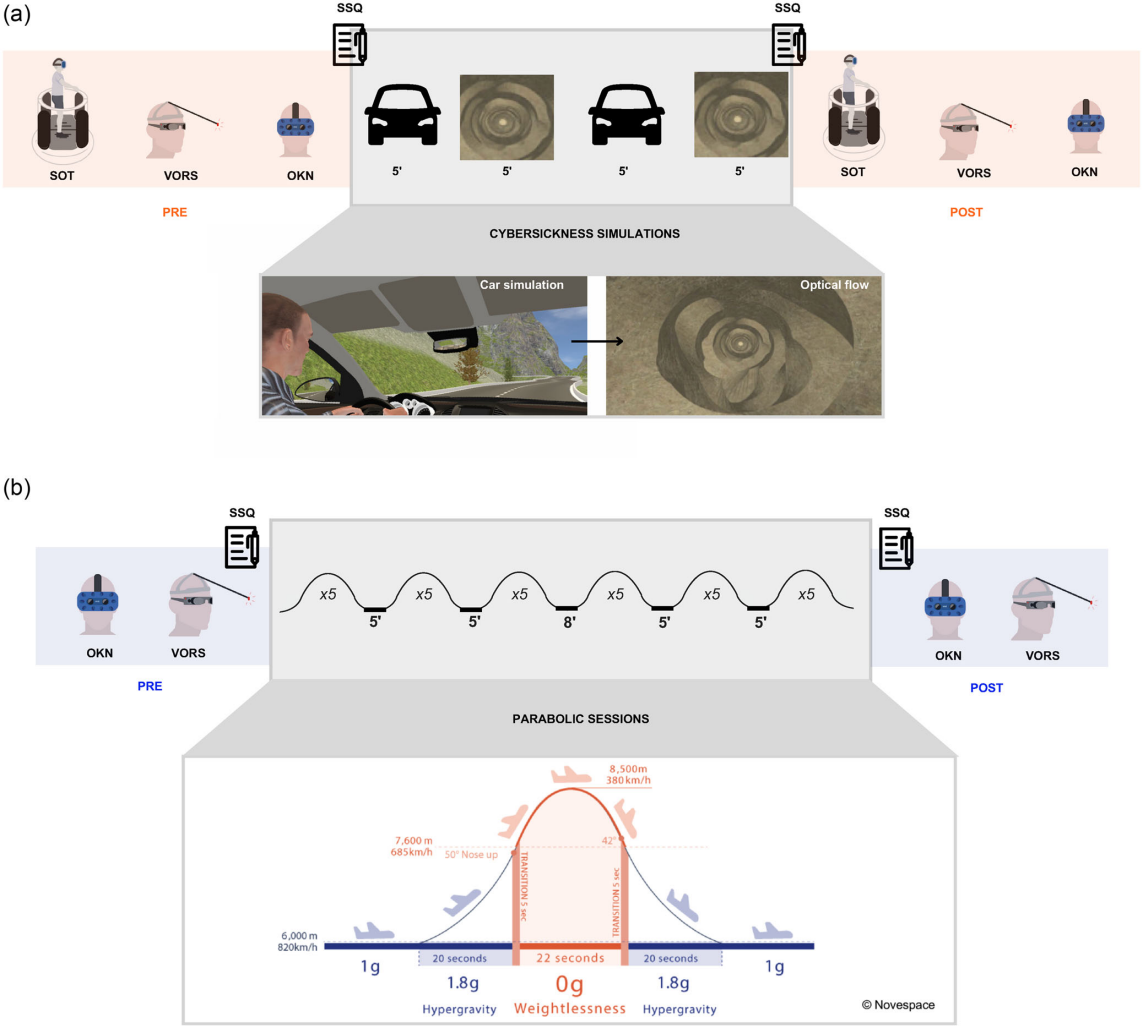
VR protocol timeline (a) and PF protocol timeline (b). The VR protocol lasted approximately 2 h, whereas the PF protocol extended to about 3 h. Times are presented in minutes. In the VR protocol, participants first completed the SOT, followed by assessments of VORS and OKN. The VR simulation consisted of two 5‐min immersive scenes, each repeated twice, or terminated earlier if participants reached a score of 4 on the Motion Sickness Severity Scale. Sensory testing was then repeated in the same order post‐exposure. In the PF, OKN and VORS were performed before the flight session and immediately after the completion of all parabolas. OKN, optokinetic nystagmus; PF, parabolic flight; SOT, sensory organization test; SSQ, simulator sickness questionnaire; VORS, vestibulo‐ocular reflex suppression; VR, virtual reality.

Vestibulo‐ocular reflexes and optokinetic nystagmus were evaluated before and after exposure to VR and to parabolic flights (Figure 1). Posturographic tests were performed in the laboratory, before and after VR exposure.

### 2.1 Ethical approval

Before taking part in the present study, all subjects provided a signed consent form after a cooling‐off period, and after inclusion visit. Ethical approval for this study was granted by the national Ethics Committee ‘Comité de protection des personnes Ile de France II’ no. 2023‐A02145‐40 and all experiments were conducted according to the *Declaration of Helsinki* (except for registration in a database).

### 2.2 Participants

Twenty‐nine healthy participants were enrolled for the project (27 ± 7 years; 14 females, 15 males). Among them, 23 participants participated in the parabolic flight (PF) protocol (28 ± 7 years; 10 females, 13 males), and 21 of them also performed the virtual reality (VR) protocol in the laboratory (27 ± 7 years; 9 females, 12 males). Six participants did only the VR protocol (22 ± 2 years; 4 females, 2 males). For participants enrolled in both VR and PF protocols, 10 did the VR protocol 1 month before the PF protocol and 11 performed the VR protocol 1 month after the PF protocol, to prevent protocol order bias. No specific sensory testing was performed prior to the experiments. None of the participants reported any health issues or previous medical history which could have impacted their sensory functions. For participants selected for the PF protocol, a medical form was filled in and approved by an aerospatial physician, based on the analysis of the participant's effort electrocardiogram made by a cardiologist.

### 2.3 Virtual reality protocol

Sensori‐motor tests were conducted in a specific order, reproduced for each participant, starting with the most comfortable test and finishing with the most provocative one. Participants first completed the Sensory Organization Test (SOT), followed by the Vestibulo‐Ocular Reflex Suppression (VORS) task, and concluded with Optokinetic Nystagmus (OKN) testing (Figure 1a). Those experimental results were accounted as baseline responses. Participants were then placed upright on a platform with a virtual reality headset for the sensory conflict procedure (MotionVR, Virtualis, Montpellier, France). They watched two different simulations: one as a front side car passenger on a mountain road, and the other inside a tunnel which rotated in a random orientation every 10 s and switched from forward to backward motion every 8 s. Each simulation lasted 5 min and was repeated twice. Continuous dialogue was maintained between the participant and the experimenter to follow the participant subjective state. The sensory conflict procedure was stopped when participants reported a moderate nausea or discomfort (Motion Sickness Severety Scale = 4; Czeiler et al., 2023) or when they requested it. Participants without symptoms remained on the simulation for a total of 20 min. Sensori‐motor evaluations were performed post‐conflict in the same order (SOT > VORS > OKN) (Figure 1a). The Simulator Sickness Questionnaire (SSQ) was filled in immediately before and after the virtual sensory conflict.

### 2.4 Parabolic flight protocol

Parabolic flights were conducted during the VP177 and VP181 parabolic flight campaigns of the Centre National d'Etudes Spatiales (CNES) aboard the Airbus A310 Zero G, operated by Novespace in Mérignac (France). Each parabolic flight consisted of 31 parabolic manoeuvres, each following a sequence of altered gravity phases: hypergravity (1.8 g), microgravity (0 g) and a return to hypergravity (1.8 g), interspersed with periods of normogravity during steady flight (Figure 1b). Each parabola lasted 1 min, with a 2‐min interval between successive parabolas. Five‐minute breaks were scheduled after every five parabolas, with an extended 8‐min break at the midpoint of the flight (Figure 1b). The total flight duration ranged from 2.5 to 3 h, encompassing takeoff, transit to the flight zone, the parabolic session, return to the airport, and landing. To prevent MS, scopolamine is commonly administered pre‐flight, as it is known to attenuate vestibular integration (Bestaven et al., 2016; Weerts et al., 2015). However, in this study, no medication was given before the flight to preserve the integrity of vestibular function and maintain participant alertness.

Before takeoff, while the aircraft was stationary on the ground, participants underwent baseline measurements of OKN (four directions, 30 s per direction, 30° s^−1^) and VORS (horizontal and vertical, each repeated twice, 30 s per trial). Additionally, VORS was performed during approximately 10 parabolas spread throughout the parabolic session (between the 5th and 25th parabola). However, the goggle‐mounted gyroscope produced incoherent kinematic data when gravity was altered, rendering the in‐flight test unusable. After the final parabola (during the steady flight before landing), OKN was tested again, whereas VORS was assessed after landing on the ground. A schematic timeline of the experimental protocol is presented in Figure 1b. Participants were allowed to withdraw from in‐flight testing or take breaks as needed. Those who were unable to continue the experiment in flight were excluded from further testing and were taken care of by the medical team.

### 2.5 Sensori‐motor tests

The optokinetic nysgtamus reflex was tested using a bilateral eye‐tracking system implemented inside a VR headset (HTC Vive ProEye, Taoyuan, Taiwan). White dots on a black background moving at 30° s^−1^ from right‐to‐left, left‐to‐right, top‐to‐bottom and bottom‐to‐top were generated by the OKN software (Virtualis; Figure 3b). The same presentation order was kept pre‐ and post‐exposure, and for each participant. Each direction recording lasted 30 s. Participants were asked to look straight forward before the start of stimulation and to let their eyes move freely without controlling them.

Vestibulo‐ocular reflex suppression was evaluated by recording the movement of the right eye pupil using an infrared camera on eye‐tracking goggles equipped with an integrated gyroscope to record head movements (Otometrics ICS Impulse, Natus, Middleton, WI, USA). To avoid visual disturbance, occulting lenses were fitted on goggles, allowing specific far‐red and infrared wavelengths to pass through. A lab‐made helmet composed of a carbon rod ending with a red LED (45 cm in front of the participant) was placed and tightened on the participant's head. The rod was adjusted to place the red target in the middle of the visual field of the participant (Figure 4a). Participants were asked by the experimenter to voluntarily move their head quickly in the horizontal or in the vertical orientation and to make a 1 s break before starting any movement in the other direction (e.g. from right‐to‐left to left‐to‐right). The task required participants to suppress the vestibulo‐ocular reflex (VOR) by keeping their eyes fixed in the head while rotating it, maintaining their gaze on a red reference LED that moved with the head. A horizontal trial was performed first and a vertical trial second, with each lasting between 30 and 40 s, with 20 head movements realized per trial. This task was repeated 3 times.

A posturographic test was conducted using the Virtualis platform (MotionVR) and its standardized sensory organization test protocol (Figure 6a). The protocol included three conditions, each comprising three 20‐s trials. The visual conditions were displayed as followed: (1) normal vision dynamically adjusted to head movements, (2) vision deprivation (black screen), and (3) fixed vision, where the visual scene remained unchanged despite head movements. These visual conditions were assessed while the platform was static. Participants were instructed to maintain a forward gaze and remain as still and stable as possible throughout each trial. Feet centre‐of‐pressure (CoP) was measured by force detectors integrated into the platform to observe posturographic variations (Figure 6a).

Vestibular‐evoked myogenic potentials (VEMP) were assessed during both protocols. This test relied on the recording of otolith‐driven muscle reflexes elicited by vestibular stimulation using tone‐burst auditory stimulation. However, no significant longitudinal effects were observed in either, and thus these results were not included in this paper.

### 2.6 Motion sickness evaluation

Subjective sensations and feelings of MS were reported using the SSQ (Kennedy et al., 1993) immediately before and after the virtual sensory conflict. For the PF protocol, SSQ was filled before the start of the first parabola and right after the end of the parabolic session.

### 2.7 Data analysis

**FIGURE 2:**
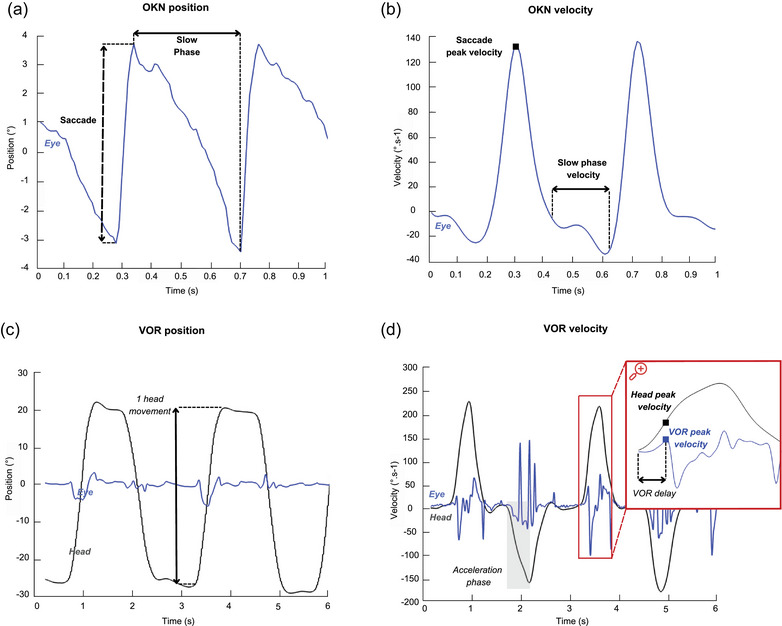
Eye position and velocity traces during OKN (a, b) and VORS (c, d) testing. For OKN (a), eye position (blue) is divided into two phases: the slow phase (continuous double‐sided arrow) where the eye tracks the moving visual stimulus, and the corrective saccade (dashed double‐sided arrow) that recaptures the visual input on the retina. Saccades between slow phases are clearly identified by velocity peaks (b). For VOR (c), both eye (blue) and head (black) movements are illustrated. Head movements are fast, large and involve a 1 s break between directional changes. The eye's corrective reflex, which opposes head movement, occurs during the head acceleration phase and represents the residual VOR response before suppression (d). Any eye movements occurring after the head acceleration phase can be caused by different strategies such as smooth pursuit or fixation. OKN, optokinetic nystagmus; VOR, vestibulo‐oculo reflex.

All analyses were performed using MATLAB R2024b (The Mathworks, Natick, MA, USA). For OKN, gaze positions were low‐pass filtered using a fourth‐order Butterworth filter with a cutoff frequency of 5 Hz (Figure 2a). Saccades were identified algorithmically by detecting velocity peaks exceeding a threshold of 100° s^−1^. The onset and offset of each saccade were determined by locating the first velocity inversions occurring before and after the velocity peak, respectively. All detected saccades where visually inspected after the algorithm was run, and mismatches were manually corrected. Eye displacements between the end of the (*n* − 1) saccade and the start of the *n*‐th saccade, characterized by lower velocity in the opposite direction, were considered slow phases (Figure 2b). Saccadic and slow phase durations and amplitudes were computed. OKN gain was calculated by dividing the eye slow‐phase velocity by the visual stimulus velocity (30° s^−1^). Nystagmus frequency was determined by dividing the total number of eye slow‐phases by the trial duration.

For VORS, vertical and horizontal eye positions in degrees and head yaw, pitch and roll (Figure 2c) were extracted, low‐pass filtered (Butterworth, fourth‐order, 20 Hz for eyes and 5 Hz for head). Velocities were then calculated by deriving the filtered signals. Head rotations were identified when head velocity peak exceed 60° s^−1^ (threshold to distinguish intentional head motion from noise), and the onset and offset of head rotation were determined by locating the first velocity inversions occurring before and after the velocity peak, respectively. The output from algorithm detection was visually inspected and manually corrected if necessary. The head acceleration phase was defined as the first part of head movement, between onset and velocity peak (Figure 2d). In the velocity plot, eye and head traces in the same direction indicate that the eyes are moving in the opposite direction compared to the head (Figure 2d). Although the residual VOR response was often weak and eye position highly variable due to refixation saccades, it was consistently observed across all head movements and participants, confirming the presence of a residual VOR as previously described by Gauthier and Vercher (1990). The task required maintaining a stable gaze on a fixation point that rotated with the head, keeping the eyes fixed relative to the head. We calculated the eye deviation variability (EDV), defined as the standard deviation of eye position during the head acceleration phase. The correction point was defined as the time at which the eye movement transitioned in the direction of the head, marking the onset of VOR suppression (Figure 2d). VOR gain was calculated as the ratio between the eye peak velocity during the remaining VOR and the head velocity at the point of correction. VOR delay was defined as the time interval between the onset of the VOR and the correction point (Figure 2d), marked by the eye moving in the direction opposite to the head.

### 2.8 Statistical analyses

**FIGURE 3:**
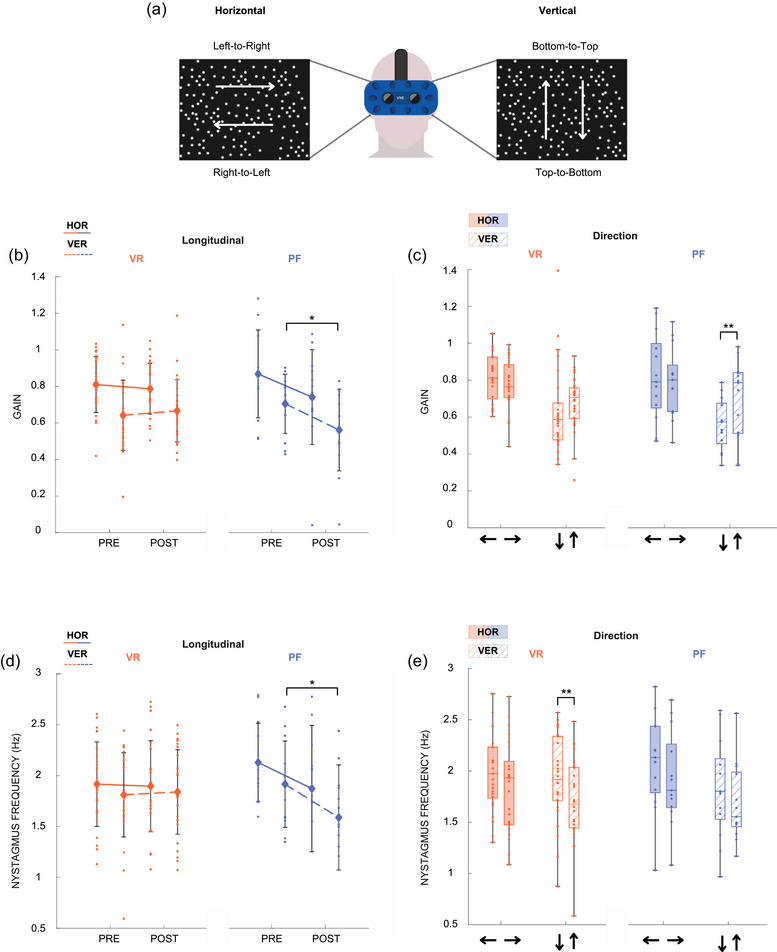
OKN results from VR and PF protocols. Directions are referred to as right‐to‐left, left‐to‐right, top‐to‐bottom, bottom‐to‐top and are described in (a). OKN gain (b, c) and nystagmus frequency (panels d, e) are presented for both paradigms and movement orientations. VR results are shown in orange, and PF results in blue. Data corresponding to horizontal movements are represented with solid fill, and vertical movements with a dashed pattern. Post‐flight, both OKN gain and nystagmus frequency were reduced in the vertical direction. Notably, bottom‐to‐top movements were facilitated compared to top‐to‐bottom, as evidenced by a higher gain in the PF condition and a lower nystagmus frequency in the VR condition. OKN gain was calculated as the ratio of eye slow‐phase velocity to visual stimulus velocity (30° s^−1^). Nystagmus frequency reflects the total number of eye slow‐phases divided by trial duration. Medians are represented within box plots. Repeated‐measure ANOVA, Fisher's LSD *post hoc*, VR: *n* = 25, PF: *n* = 13, *P < 0.05, **P < 0.01. HOR, horizontal direction; OKN, optokinetic nystagmus; PF, parabolic flight; VER, vertical direction; VR, virtual reality.

**FIGURE 4:**
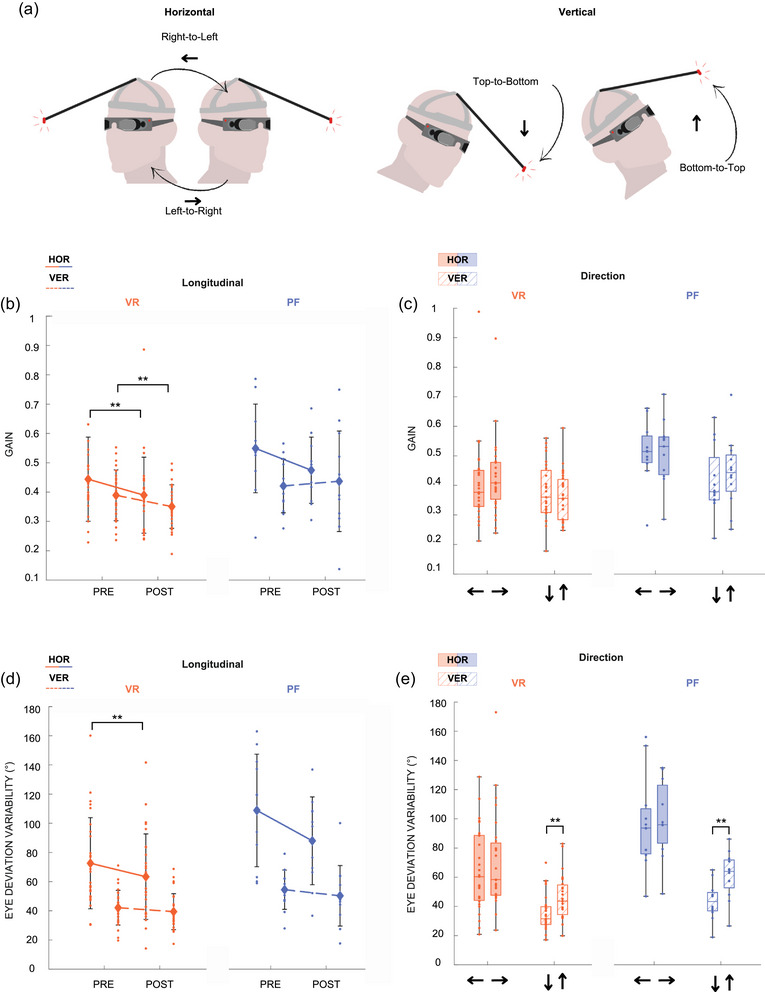
VORS results from VR and PF protocols. Directions are referred to as right‐to‐left, left‐to‐right, top‐to‐bottom, bottom‐to‐top and are described in (a). Gain‐at‐correction (b, c) and eye deviation variability (d, e) are shown for both paradigms and orientations. VR results are shown in orange, and PF results in blue. Data corresponding to horizontal movements are represented with solid fill, and vertical movements with a dashed pattern. Post‐VR, VOR gain decreased in both directions, while only horizontal eye deviation variability was reduced. A vertical asymmetry in eye deviation variability was observed in both paradigms, with greater variability during bottom‐to‐top movements. VOR gain was calculated as the ratio between the eye peak velocity during the remaining VOR and the head velocity at the point of correction. EDV was defined as the standard deviation of eye position during the head acceleration phase. Medians are represented within box plots. Repeated‐measure ANOVA, Fisher's LSD *post hoc*, VR: *n* = 27, PF: *n* = 11–12, *P < 0.05, **P < 0.01. EDV, eye deviation variability; HOR, horizontal direction; PF, parabolic flight; VER, vertical direction; VOR, vestibulo‐ocular reflex; VR, virtual reality.

Statistical analyses were conducted using MATLAB for repeated‐measures ANOVA and JASP for correlation and Wilcoxon's signed‐rank test. Pre–post and directional effects were assessed separately for horizontal and vertical data. For both on‐ground and in‐flight results, we performed repeated‐measures ANOVA with Fisher's least significant difference (LSD) correction. Prior to running the ANOVA, Levene's test was used to verify homogeneity of variance, which was satisfied in all analyses (*P* > 0.05). Mauchly's test of sphericity was also conducted, and if the assumption was violated (*P* < 0.05), the Greenhouse–Geisser correction was applied when epsilon (ε) was <0.75, whereas the Huynh–Feldt correction was used for ε ≥ 0.75. Effect sizes for ANOVA results were reported as partial eta squared (η^2^), calculated as the ratio of the effect sum of squares to the sum of the effect and error sum of squares. Correlation analyses were conducted using Spearman's ρ. SOT pre–post effects were tested using a non‐parametric Wilcoxon's signed‐rank test, based on the Shapiro–Wilk normality test results. Effect size was reported as matched rank biserial correlation (*r*) value. Results are presented as means ± standard deviation (SD) unless otherwise specified within the text, whereas medians are represented in box plots within Figures 3 and 4.

## 3 RESULTS

We assessed OKN and VOR performance before and after exposure to virtual reality or parabolic flight to evaluate longitudinal effects on the reflexes. Initial descriptions of the responses are provided, followed by a detailed presentation of significant results.

### 3.1 Optokinetic nystagmus

#### 3.1.1 Virtual reality

In the VR protocol, baseline OKN amplitudes were 7.9 ± 3.7° (horizontal) and 7.1 ± 3.7° (vertical), with corresponding durations of 322 ± 117 ms and 333 ± 138 ms. The mean slow‐phase velocity was 24 ± 4.6° s^−1^ for horizontal movements and 19.8 ± 5.8° s^−1^ for vertical. Nystagmus frequency was approximately 1.9 ± 0.5 Hz in both directions.

The only significant effect observed was a directional difference in nystagmus frequency, with top‐to‐bottom movements eliciting higher nystagmus than bottom‐to‐top (TtoB = 1.92 ± 0.48 Hz, BtoT = 1.73 ± 0.44 Hz, *F*(1,25) = 8.6, *P* = 0.00714, η^2^ = 0.26) (Table 1, line 8; Figure 3e). No pre–post effects were found in any of the OKN tested parameters.

**TABLE 1:** Statistical analysis of OKN parameters in VR and PF.

Test	Session	Variable	Effect	Orientation	Level 1	Level 2	*F*	*P*	η^2^	Line
**OKN**	**VR**	Gain	Longitudinal	Horizontal	Pre: 0.80 ± 0.18	Pos: 0.79 ± 0.16	(1,24) = 0.50	0.483	n.a.	1
				Vertical	Pre: 0.64 ± 0.21	Post: 0.67 ± 0.20	(1,25) = 1.20	0.282	n.a.	2
			Direction	Horizontal	RtoL: 0.82 ± 0.16	LtoR: 0.77 ± 0.18	(1,24) = 3.55	0.0717	n.a.	3
				Vertical	TtoB: 0.64 ± 0.24	BtoT: 0.67 ± 0.16	(1,25) = 1.25	0.273	n.a.	4
		Nystagmus frequency (Hz)	Longitudinal	Horizontal	Pre: 1.92 ± 0.54	Post: 1.90 ± 0.53	(1,24) = 0.004	0.949	n.a.	5
				Vertical	Pre: 1.82 ± 0.48	Post: 1.84 ± 0.46	(1,25) = 0.18	0.674	n.a.	6
			**Direction**	Horizontal	RtoL: 1.99 ± 0.43	LtoR: 1.76 ± 0.6	(1,24) = 0.7	0.410	n.a.	7
				**Vertical**	**TtoB: 1.92 ± 0.48**	**BtoT: 1.73 ± 0.44**	**(1,25) = 8.6**	0.00714*	**0.26**	8
		Slow phase amplitude (°)	Longitudinal	Horizontal	Pre: 7.88 ± 3.92	Post: 7.94 ± 3.48	(1,24) = 0.15	0.704	n.a.	9
				Vertical	Pre: 6.94 ± 3.56	Post: 7.31 ± 3.92	(1,25) = 0.66	0.425	n.a.	10
			Direction	Horizontal	RtoL: 7.37 ± 2.50	LtoR: 8.48 ± 4.56	(1,24) = 2.90	0.102	n.a.	11
				Vertical	TtoB: 6.91 ± 4.31	BtoT: 7.34 ± 3.05	(1,25) = 1.08	0.310	n.a.	12
		Slow phase duration (ms)	Longitudinal	Horizontal	Pre: 550 ± 1660	Post: 320 ± 120	(1,24) = 0.94	0.341	n.a.	13
				Vertical	Pre: 640 ± 2330	Post: 350 ± 150	(1,25) = 0.88	0.357	n.a.	14
			Direction	Horizontal	RtoL: 301 ± 109	LtoR: 576 ± 1673	(1,24) = 1.37	0.253	n.a.	15
				Vertical	TtoB: 318 ± 166	BtoT: 671 ± 2343	(1,25) = 1.28	0.268	n.a.	16
	**PF**	Gain	**Longitudinal**	Horizontal	Pre: 0.80 ± 0.18	Post: 0.78 ± 0.16	(1,12) = 2.50	0.139	n.a.	17
				**Vertical**	**Pre: 0.72 ± 0.19**	**Post: 0.59 ± 0.23**	**(1,12) = 7.80**	0.0164*	**0.39**	18
			**Direction**	Horizontal	RtoL: 0.81 ± 0.29	LtoR: 0.78 ± 0.22	(1,12) = 0.54	0.475	n.a.	19
				**Vertical**	**TtoB: 0.58 ± 0.18**	**BtoT: 0.72 ± 0.23**	**(1,12) = 15.8**	0.00186*	**0.57**	20
		Nystagmus frequency (Hz)	**Longitudinal**	Horizontal	Pre: 2.13 ± 0.47	Post: 2.01 ± 0.58	(1,12) = 2.70	0.127	n.a.	21
				**Vertical**	**Pre: 1.83 ± 0.46**	**Post: 1.69 ± 0.51**	**(1,12) = 5.70**	0.0346*	**0.32**	22
			Direction	Horizontal	RtoL: 2.06 ± 0.57	LtoR: 1.95 ± 0.52	(1,12) = 3.06	0.106	n.a.	23
				Vertical	TtoB: 1.80 ± 0.54	BtoT: 1.66 ± 0.44	(1,12) = 2.10	0.177	n.a.	24
		Slow phase amplitude (°)	Longitudinal	Horizontal	Pre: 8.34 ± 4.57	Post: 6.89 ± 2.97	(1,12) = 2.65	0.129	n.a.	25
				Vertical	Pre: 7.91 ± 4.56	Post: 6.67 ± 3.06	(1,12) = 1.81	0.203	n.a.	26
			**Direction**	Horizontal	RtoL: 7.60 ± 3.72	LtoR: 7.63 ± 4.08	(1,12) = 3.16	0.101	n.a.	27
				**Vertical**	**TtoB: 5.95 ± 3.70**	**BtoT: 8.39 ± 3.79**	**(1,12) = 22.0**	<0.001*	**0.65**	28
		Slow phase duration (ms)	Longitudinal	Horizontal	Pre: 280 ± 110	Post: 510 ± 790	(1,12) = 1.23	0.290	n.a.	29
				Vertical	Pre: 360 ± 130	Post: 520 ± 580	(1,12) = 1.69	0.217	n.a.	30
			Direction	Horizontal	RtoL: 369 ± 505	LtoR: 415 ± 625	(1,12) = 3.87	0.0726	n.a.	31
				Vertical	TtoB: 430 ± 510	BtoT: 440 ± 340	(1,12) = 0.35	0.564	n.a.	32

Rows 1–8 correspond to VR data, and rows 9–16 to PF data. Longitudinal and directional effects are reported for OKN gain, nystagmus frequency, slow phase amplitude and duration. Repeated‐measure ANOVA, Fisher's LSD *post hoc*, VR: *n* = 25, PF: *n *= 13, *p < 0.05; **p < 0.01. Lines in bold correspond to significant effects.

#### 3.1.2 Parabolic flight

In the PF protocol, OKN amplitudes were 7.6 ± 3.9° (horizontal) and 7.3 ± 3.9° (vertical). The average duration was 294 ± 102 ms for horizontal and 367 ± 128 ms for vertical. Horizontal eye slow‐phase velocity reached 24.6 ± 6.4° s⁻¹, while vertical velocity was 20.3 ± 5.7° s⁻¹. Nystagmus frequency averaged 2.0 ± 0.5 Hz in the horizontal and 1.7 ± 0.5 Hz in the vertical direction.

The pre–post effect was significant for both OKN gain and nystagmus frequency, but only in the vertical direction. Post‐flight, participants exhibited reduced vertical OKN gain (PRE = 0.72 ± 0.19, POST = 0.59 ± 0.23, *F*(1,12) = 7.8, *P* = 0.0164, η^2^ = 0.39) and decreased vertical nystagmus frequency (PRE = 1.83 ± 0.46 Hz, POST = 1.69 ± 0.51 Hz, *F*(1,12) = 5.7, *P* = 0.0346, η^2^ = 0.32) (Table 1, lines 18 and 22; Figure 3b, d). A directional effect was also observed, with bottom‐to‐top gain (TtoB = 0.58 ± 0.18, BtoT = 0.72 ± 0.23, *F*(1,12) = 15.8, *P* = 0.00186, η^2^ = 0.57) and slow‐phase amplitude (TtoB = 5.95 ± 3.70°, BtoT = 8.39 ± 3.79°, *F*(1,12) = 22.0, *P* < 0.001, η^2^ = 0.65) being higher compared to top‐to‐bottom (Table 1, lines 20 and 28; Figure 3c). Unlike the VR protocol, no directional effect was observed in vertical nystagmus frequency (Table 1, line 24; Figure 3e).

### 3.2 Vestibulo‐ocular reflex suppression

#### 3.2.1 Virtual reality

During the VR protocol, horizontal head movements had a mean amplitude of 79 ± 19° with a duration of 671 ± 118 ms while vertical movements were 57 ± 14° and lasted 746 ± 146 ms. Peak head velocities were 389 ± 154° s⁻¹ (horizontal) and 260 ± 79° s⁻¹ (vertical), with corresponding peak accelerations of 2135 ± 1233° s⁻^2^ (horizontal) and 1391 ± 611° s⁻^2^ (vertical).

Voluntary head movements were consistent post‐VR exposure, with no differences in peak head velocity either horizontally (PRE = 386 ± 162° s⁻¹, POST = 391 ± 147° s⁻¹; *F*(1,26) = 0.17, *P* = 0.687) or vertically (PRE = 261 ± 80° s⁻¹, POST = 259 ± 79° s⁻¹; *F*(1,26) = 0.06, *P* = 0.803) (Table 2, lines 1 and 2). Similarly, peak head acceleration remained stable, with no significant change, confirming that head movements remained consistent across both time points in the VR protocol (Horizontal: PRE = 2136 ± 1288° s⁻^2^, POST = 2134 ± 1188° s⁻^2^; *F*(1,26) = 0.0003, *P* = 0.987; Vertical: PRE = 1409 ± 623° s⁻^2^, POST = 1372 ± 604° s⁻^2^; *F*(1,26) = 0.46, *P* = 0.505) (Table 2, lines 3 and 4).

**TABLE 2:** Statistical analysis of VOR parameters in VR and PF.

Test	Session	Variable	Effect	Orientation	Level 1	Level 2	*F*	*P*	η^2^	Line
***VORS***	**VR**	Head peak velocity (° s⁻¹)	Longitudinal	Horizontal	Pre: 386 ± 162	Post: 391 ± 147	(1,26) = 0.17	0.687	n.a.	1
				Vertical	Pre: 261 ± 80	Post: 259 ± 79	(1,26) = 0.06	0.803	n.a.	2
		Head peak acceleration (° s‐2)	Longitudinal	Horizontal	Pre: 2136 ± 1288	Post: 2134 ± 1188	(1,26) = 0.0003	0.987	n.a.	3
				Vertical	Pre: 1409 ± 623	Post: 1372 ± 604	(1,26) = 0.46	0.505	n.a.	4
		Gain	**Longitudinal**	**Horizontal**	**Pre: 0.44 ± 0.15**	**Post: 0.39 ± 0.13**	**(1,26) = 13.2**	0.00120*	**0.34**	5
				**Vertical**	**Pre: 0.39 ± 0.10**	**Post: 0.35 ± 0.09**	**(1,26) = 12.2**	0.00176*	**0.32**	6
			Direction	Horizontal	RtoL: 0.38 ± 0.15	LtoR: 0.41 ± 0.14	(1,26) = 1.90	0.180	n.a.	7
				Vertical	TtoB: 0.38 ± 0.10	BtoT: 0.36 ± 0.09	(1,26) = 0.52	0.477	n.a.	8
		Eye Deviation Variability (°)	**Longitudinal**	**Horizontal**	**Pre: 72.6 ± 33**	**Post: 63.4 ± 29.4**	**(1,26) = 11.4**	0.00235*	**0.30**	9
				Vertical	Pre: 42.1 ± 15.5	Post: 39.4 ± 16.1	(1,26) = 3.90	0.0601	n.a.	10
			**Direction**	Horizontal	RtoL: 62.7 ± 29.6	LtoR: 64.6 ± 33.3	(1,26) = 5.10	0.170	n.a.	11
				**Vertical**	**TtoB: 35.2 ± 13.5**	**BtoT: 46.3 ± 16.0**	**(1,26) = 13.9**	<0.001*	**0.35**	12
		VOR delay (ms)	Longitudinal	Horizontal	Pre: 183 ± 52	Post: 185 ± 63	(1,26) = 0.10	0.753	n.a.	13
				Vertical	Pre: 224 ± 74	Post: 237 ± 77	(1,26) = 2.70	0.110	n.a.	14
			Direction	Horizontal	RtoL: 189.2 ± 56.5	LtoR: 179.0 ± 58.5	(1,26) = 0.786	0.383	n.a.	15
				Vertical	TtoB: 226.5 ± 80.4	BtoT: 233.1 ± 70.5	(1,26) = 0.240	0.629	n.a.	16
	**PF**	Head peak velocity (° s⁻¹)	Longitudinal	Horizontal	Pre: 488 ± 96	Post: 502 ± 123	(1,10) = 0.01	0.914	n.a.	17
				Vertical	Pre: 347 ± 67	Post: 354 ± 83	(1,11) = 0.04	0.849	n.a.	18
		Head peak acceleration (° s‐2)	Longitudinal	Horizontal	Pre: 3096 ± 986	Post: 3360 ± 1229	(1,10) = 0.12	0.731	n.a.	19
				Vertical	Pre: 2330 ± 697	Post: 2474 ± 857	(1,11) = 0.14	0.712	n.a.	20
		Gain	Longitudinal	Horizontal	Pre: 0.54 ± 0.15	Post: 0.47 ± 0.12	(1,10) = 2.40	0.151	n.a.	21
				Vertical	Pre: 0.42 ± 0.10	Post: 0.44 ± 0.21	(1,11) = 0.07	0.799	n.a.	22
			Direction	Horizontal	RtoL: 0.54 ± 0.14	LtoR: 0.53 ± 0.14	(1,10) = 0.004	0.951	n.a.	23
				Vertical	TtoB: 0.40 ± 0.17	BtoT: 0.44 ± 0.16	(1,11) = 0.37	0.557	n.a.	24
		Eye Deviation Variability (°)	Longitudinal	Horizontal	Pre: 108.8 ± 39.3	Post: 88 ± 30	(1,10) = 3.10	0.106	n.a.	25
				Vertical	Pre: 54.5 ± 18.6	Post: 50.3 ± 24.8	(1,11) = 0.40	0.541	n.a.	26
			**Direction**	Horizontal	RtoL: 95.9 ± 39.1	LtoR: 98.4 ± 33.2	(1,10) = 0.21	0.658	n.a.	27
				**Vertical**	**TtoB: 43.6 ± 14.5**	**BtoT: 61.2 ± 24.5**	**(1,11) = 26**	<0.001*	**0.70**	28
		VOR delay (ms)	Longitudinal	Horizontal	Pre: 139 ± 36	Post: 134 ± 42	(1,10) = 0.02	0.883	n.a.	29
				Vertical	Pre: 158 ± 38	Post: 157 ± 52	(1,11) = 0.006	0.939	n.a.	30
			Direction	Horizontal	RtoL: 130.8 ± 33.2	LtoR: 141.9 ± 44.0	(1,10) = 1.99	0.189	n.a.	31
				Vertical	TtoB: 158.9 ± 47.9	BtoT: 156.4 ± 43.3	(1,11) = 0.14	0.713	n.a.	32

Rows 1–6 correspond to VR data, and rows 7–12 to PF data. Longitudinal and directional effects are reported for VOR gain, eye deviation variability and VOR delay. Repeated‐measure ANOVA, Fisher's LSD *post hoc*, VR: *n *= 27, PF: *n *= 11–12, *p < 0.05; **p < 0.01. Lines in bold correspond to significant effects.

A significant longitudinal effect was observed in VOR gain, with a significant decrease after VR exposure in both horizontal (PRE = 0.44 ± 0.15, POST = 0.39 ± 0.13, *F*(1,26) = 13.2, *P* = 0.00120, η^2^ = 0.34) and vertical orientations (PRE = 0.39 ± 0.1, POST = 0.35 ± 0.09, *F*(1,26) = 12.2, *P* = 0.00176, η^2^ = 0.32) (Table 2, lines 5 and 6; Figure 4b). Eye deviation variability was significantly reduced post‐exposure in the horizontal direction (PRE = 72.6 ± 33°, POST = 63.4 ± 29.4°, *F*(1,26) = 11.4, *P* = 0.00235, η^2^ = 0.30) (Table 2, line 9; Figure 4d). A directional effect was found in the vertical direction, with bottom‐to‐top movements eliciting higher eye deviation variability compared to top‐to‐bottom (TtoB = 35.2 ± 13.5°, BtoT = 46.3 ± 16.0°, *F*(1,26) = 13.9, *P* < 0.001, η^2^ = 0.35) (Table 2, line 12; Figure 4e). No significant effect was found in response delay in this protocol (Table 2, lines 13–16; Figure 4c).

#### 3.2.2 Parabolic flight

In the PF protocol, horizontal head movements averaged 72 ± 12° and lasted 538 ± 75 ms. In the vertical orientation, movements were 53 ± 12° with a duration of 572 ± 95 ms. Peak head velocities were 499 ± 109° s⁻¹ (horizontal) and 350 ± 74° s⁻¹ (vertical), with corresponding peak head acceleration of 3254 ± 1119° s⁻^2^ and 2402 ± 776° s⁻^2^, respectively.

Head movement kinematics were stable post‐flight, with no differences in peak head velocity in horizontal (PRE = 488 ± 96° s⁻¹, POST = 502 ± 123° s⁻¹, *F*(1,10) = 0.01, *P* = 0.914) and vertical direction (PRE = 347 ± 67° s⁻¹, POST = 354 ± 83° s⁻¹, *F*(1,11) = 0.04, *P* = 0.849) (Table 2, lines 17 and 18). Likewise, no significant pre–post differences were found in peak head acceleration, confirming that head movements remained consistent across both time points in the PF protocol (Horizontal: PRE = 3096 ± 986° s⁻^2^, POST = 3360 ± 1229° s⁻^2^, *F*(1,10) = 0.12, *P* = 0.731; Vertical: PRE = 2330 ± 697° s⁻^2^, POST = 2474 ± 857° s⁻^2^, *F*(1,11) = 0.14, *P* = 0.712) (Table 2, lines 19 and 20).

A single significant effect was observed in the vertical direction for eye deviation variability, where, similar to the VR protocol, bottom‐to‐top movements induced higher eye deviation variability than top‐to‐bottom (TtoB = 43.6 ± 14.5, BtoT = 61.2 ± 24.5, *F*(1,11) = 26, *P* < 0.001, η^2^ = 0.70) (Table 2, line 28; Figure 4d). No longitudinal effects were reported.

#### 3.2.3 Posturography and VR

**FIGURE 5:**
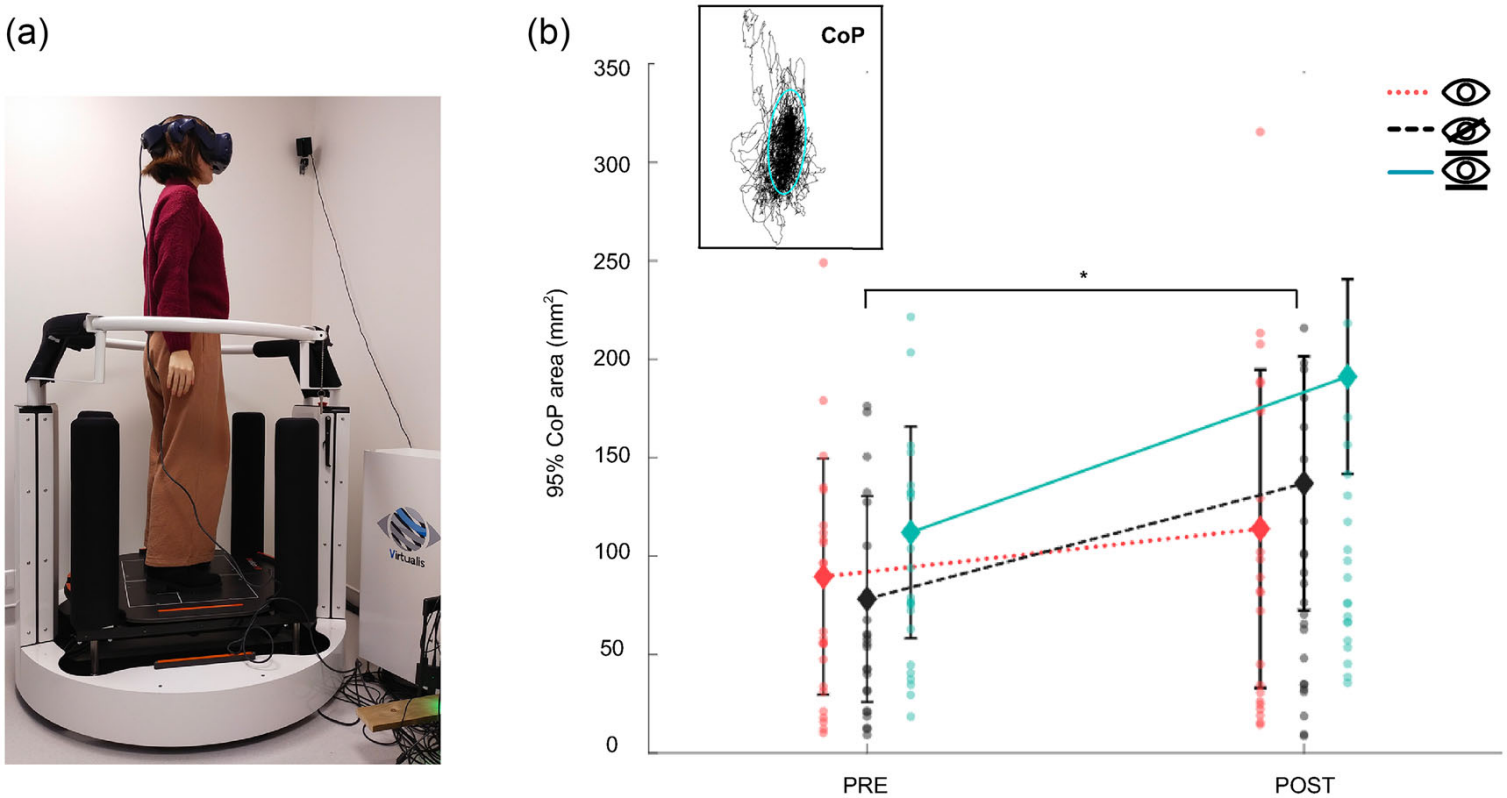
SOT results before and after VR sensory conflict. (a) The SOT set‐up. (b) The feet CoP with the three experimental conditions presented as dotted line for normal visual input, dashed line for vision deprivation, and filled line for fixed visual environment despite head movements. The platform measuring the CoP remained stable during all these conditions. Post‐VR, postural instability was increased in the deprived vision condition. The 95% area represents the elliptical region (red ellipse, b) encompassing 95% of the CoP displacement during the trials. Statistical significance was assessed using non‐parametric Wilcoxon signed‐rank test (*n* = 27). Significance levels are indicated as follows: *P < 0.05, **P < 0.01. CoP, centre‐of‐pressure; SOT, sensory organisation test; VR, virtual reality.

The 95% area of the centre‐of‐pressure (CoP) displacement was used to assess postural instability, representing the total CoP displacement area during each trial (Figure 5a).

A significant effect of VR exposure was observed, with an increase of 95% area in all three conditions. This increase was significant in the vision deprivation condition (PRE = 78.2 ± 67 mm^2^, POST = 136.9 ± 174.6 mm^2^, *W* = 86, *Z* = −2.058, *P* = 0.020, *r* = −0.471) (Figure 5b) whereas a trend was noted in the fixed vision condition (Table 3, lines 1 and 3; Figure 5b). This suggests that vestibular processing is impaired after VR exposure, an effect that becomes more apparent when visual compensation is unavailable.

**TABLE 3:** Statistical analysis of SOT parameters.

Test	Variable	Effect	Orientation	Level 1	Level 2	*W*	*Z*	*P*	*r*	Line
***Posturography***	CoP 95% area	**Longitudinal**	Normal vision	Pre: 89.6 ± 70.8	Post: 113.9 ± 97.4	124	−1.036	0.156	0.237	1
			**Deprived vision**	**Pre: 78.2 ± 67**	**Post: 136.9 ± 174.6**	**86**	**−2.058**	**0.020**	**−0.471**	2
			Fixed vision	Pre: 112 ± 91	Post: 191.2 ± 237.1	105	−1.547	0.063	−0.354	3

Longitudinal effects are reported for 95% area of CoP in three visual conditions: Normal, deprived and fixed. Wilcoxon signed‐rank test, effect size: matched rank biserial correlation (*r*) value, *n* = 27, *p < 0.05; **p < 0.01. Lines in bold correspond to significant effects.

In conclusion, absence of visual information might favour postural instability post‐VR exposure via potential modifications of vestibulo‐proprioceptive integration.

#### 3.2.4 Sensorimotor performance and motion sickness susceptibility

As previous findings showed that the type of sensory conflict implies a specific modulation of sensory integration, we hypothesized that specific sensory sensitivity might be involved in susceptibility to specific motion sickness.

Correlation analyses were performed to examine the relationships between sensorimotor variables and subjective MS scores, aiming to identify potential predictors of MS. For the VR protocol, we hypothesized that horizontal OKN might correlate with nausea scores, as virtual environments predominantly involve horizontal visual motion. In contrast, since perturbations in the PF protocol were primarily vertical and detected by the vestibular system, we proposed that this orientation would be more relevant for assessing correlations between VOR parameters and nausea scores. In both correlation tests, we examined gain from OKN testing and EDV from VORS testing, as these two variables exhibited longitudinal changes after exposure to either sensory conflict paradigm.

**FIGURE 6:**
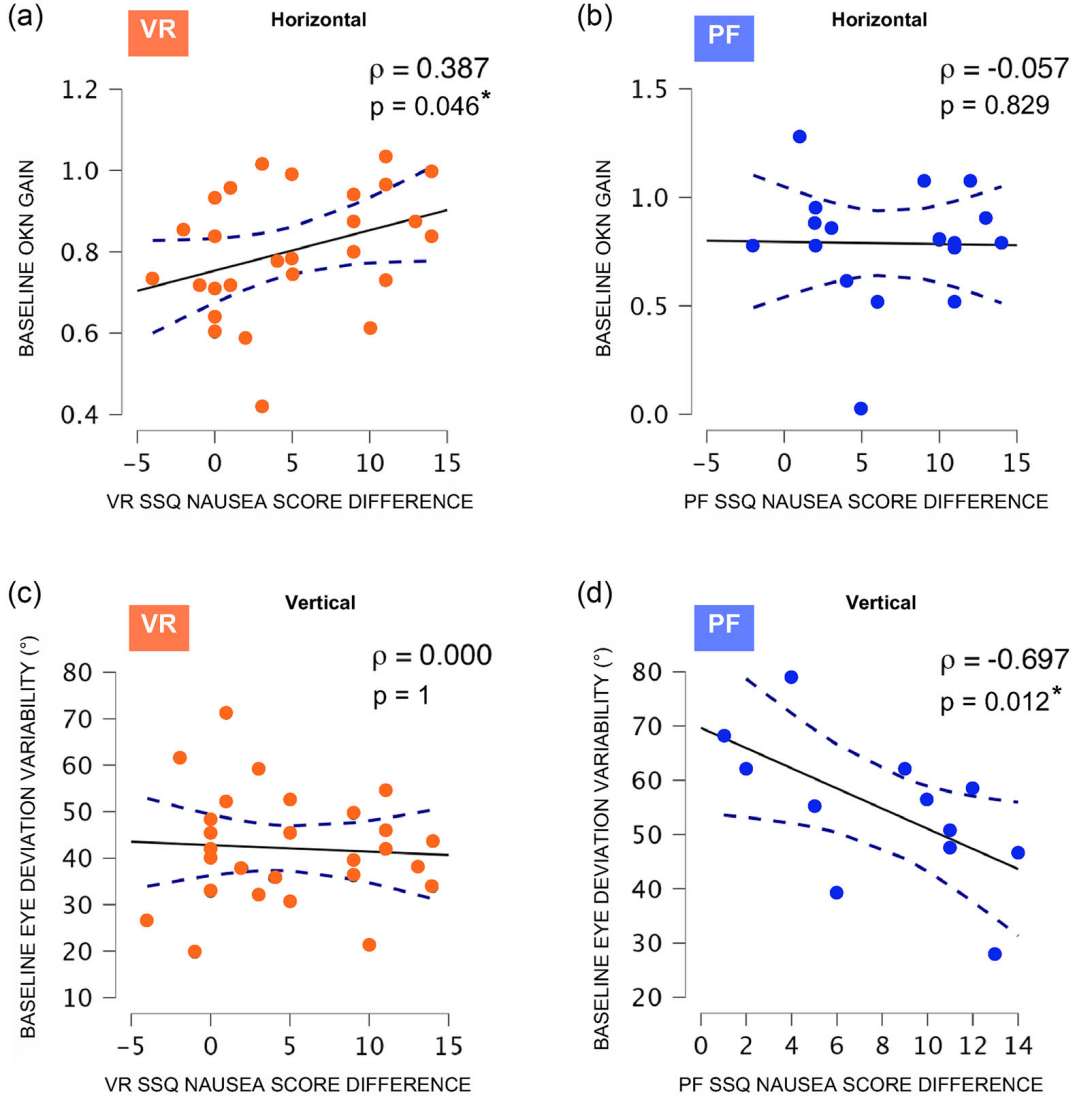
Correlations between sensory performance and motion sickness severity. Correlations between SSQ nausea score differences and OKN gain (a, b), and between SSQ nausea score differences and baseline eye deviation variability (c, d) are presented.  Baseline eye deviation variability in parabolic flight refers to pre‐flight values measured before take‐off. Severity of experienced nausea in VR was correlated with higher horizontal baseline OKN gain, whereas greater nausea symptoms post‐flight was related to lower vertical baseline eye instability. Spearman correlations. VR: *n* = 27, PF: *n* = 12–17, ρ < 0.3 low, ρ = 0.3–0.5 moderate, ρ > 0.5 high. PF, parabolic flight; SSQ, simulator sickness questionnaire; VR, virtual reality.

In the VR protocol, baseline horizontal OKN gain showed a significant positive correlation with SSQ nausea score (ρ = 0.387, *n* = 27, *P* = 0.046) (Figure 6a). Higher horizontal OKN sensitivity resulted in greater nausea symptoms post‐VR within our participants. However, baseline vertical EDV measured during the VORS test was not significantly correlated with changes in SSQ scores across the VR paradigm (Figure 6c).

In contrast, in the PF protocol, a significant negative correlation was found between vertical baseline EDV and SSQ score changes (ρ = −0.697, *n* = 12, *P* = 0.012) (Figure 6d). Lower vertical eye instability was associated with greater post‐flight nausea symptoms in participants. No significant correlations were found between baseline horizontal OKN gain and SSQ score changes in the PF protocol (Figure 6b).

In conclusion, visuo‐oculomotor sensitivity may be associated with cybersickness, while measures reflecting VORS appear more closely related to SMS susceptibility.

## 4 DISCUSSION

In this paper, we have explored sensory modulations following a brief, single exposure to two distinct MS inducing paradigms. Our findings provide human evidence of sensory reweighting in low‐level behavioural reflexes after exposure to sensory conflicts. Specifically, we observed a decreased OKN performance after parabolic flight, and an improved suppression of the VOR following virtual reality exposure. We demonstrate that cybersickness and space‐sickness have differential effects on visual and vestibular integration. Moreover, our correlation analyses suggest that MS susceptibility is influenced by an individual's sensitivity to the sensory cues most involved in detecting displacement. Cybersickness susceptibility was associated with horizontal optokinetic gain, whereas space motion sickness was linked to the robustness of the vestibulo‐ocular reflex.

Both sensory tests produced results consistent with the literature. Baseline OKN gain (∼0.8) and eye velocity (20–30° s^−1^) matched previous findings (Kanari et al., 2017; Watanabe et al., 1986). VOR suppression began 100–200 ms after head acceleration onset, with pre‐suppression gain around 0.1–0.4 – values in line with prior studies (Gauthier & Vercher, 1990; Jacobson et al., 2012; Jell et al., 1988).

The nature of the sensory cues used to induce sensory conflicts influences the subsequent sensory reweighting that occurs after exposure. We initially hypothesized that the stimulated sensory modality would exhibit diminished function after exposure to the respective sensory conflicts. Surprisingly, this was not the case, as in both types of exposure, the sensory cue considered most reliable appeared to be the one that was stimulated. The primary reflex affected by the vestibular challenge of parabolic flight was visually driven, as shown by a post‐flight decrease in OKN gain. During the flight, visual cues within the cabin remain relatively stable, while otolithic inputs signal a large altitude change (∼8000 feet). The otolithic modality is the most stimulated while the visual information is perceived as unreliable and consequently downweighted. Moreover, otolithic signals align with non‐vestibular graviceptors and proprioceptive inputs (Mittelstaedt, 1996; von Gierke & Parker, 1994), further reinforcing a sensory weighting shift that selectively affects OKN. Moreover, we anticipated an otolitho‐canalar conflict under modified gravitational conditions. In such environments, any head rotation along an axis misaligned with the gravitational field generates a discrepancy between the rotational acceleration detected by the semicircular canals and the signal from the otolith sensors. The expected variation in otolithic stimulation differs from what occurs on Earth for a similar semicircular‐detected head rotational acceleration. According to the neural mismatch theory, this otolitho‐canalar incongruence – relative to the Earth‐learned ratio ‐ could be a key factor in SMS. Based on this framework, we hypothesized that the reliability of vestibular cues in general would be questioned in such situations, leading to a decrease in vestibular weighting. However, since the measured VOR showed little to no alteration post‐flight, this hypothesis is not strongly supported, suggesting that otolitho‐canalar integration in modified gravity might not generate neural mismatch. A possible explanation is that otolithic angular detection relies on the ratio between utricular and saccular signals, and this ratio remains unaffected by gravity, which influences both utricular and saccular signals in a similar manner. In this case, it would not generate a conflict with semicircular canal information. However, no studies in the current literature have explicitly investigated or confirmed this hypothesis.

In parabolic flight, the otolithic inputs represent a novel and dynamic perturbation, triggering a reweighting of sensory integration to prioritize different sensory cues. On the other hand, in the VR protocol, the absence of vestibular detection of motion was perceived as dysfunction rather than perturbation. Only vestibular‐driven reflexes are altered following exposure to the visual challenge induced by VR, with a reduction in VOR gain observed post‐exposure. Additionally, postural stability decreases after VR exposure in the eyes‐closed condition, that is, when balance depends solely on proprioceptive and vestibular inputs. These findings confirm that vestibular performance is impaired following a visually induced sensory conflict. This outcome was unexpected, as vestibular and proprioceptive inputs remained consistent, while only the visual system signalled motion (vection). We initially hypothesized that visual information would again be downweighted. However, the absence of coherent accelerometric cues to support the perceived visual motion appeared to reduce reliance on vestibular inputs instead. Thus, in this protocol, sensory conflicts involving inactive modalities lead to a decreased contribution from those inputs. Importantly, our results align with the framework of multisensory integration based on Bayesian inference, whereby the nervous system downweights less reliable sensory modalities to minimize perceptual uncertainty (Knill & Pouget, 2004). Specifically, we observed a reduced influence of the less‐stimulated modality – vestibular inputs on the ground, and visual inputs during flight ‐.

Other studies have investigated adaptive integrative processes through repetitive exposure and habituation protocols. Morgan and colleagues (2008) demonstrated that neurons in macaque MSTd modulate the weighting of visual information, increasing reliance on vestibular inputs when visual cues are degraded. Similarly, in mice, a 2‐week visuo‐vestibular conflict led to reduced VOR performance, accompanied by decreased synaptic efficiency in the vestibular nuclei (Idoux et al., 2018). In humans, a study examining MS reduction through a 5‐day habituation protocol to visuo‐vestibular conflict found that participants experienced shortened vestibular time constants and a decrease in MS symptoms during provocative tests (Dai et al., 2011). These studies are based on recordings conducted either after long‐term habituation or during sensory conflict. Unlike the studies cited, the reweighting observed in our protocols occurred following unique or short repetitive exposure, even though neither visual nor vestibular cues were degraded during the post‐test phase. This finding highlights an adaptive mechanism wherein a brief exposure of just 20 min was sufficient to induce sensory reweighting. Notably, this reweighting was observed in low‐level motor reflexes, contrasting with earlier human studies that primarily used perceptual protocols requiring higher cognitive processing (Alais & Burr, 2004). This suggests that adaptation occurs at an early stage of sensory integration, such as within the vestibular nucleus in the case of the VOR.

The divergence in reflex effects across paradigms raises critical questions, as these reflexes share common anatomical pathways. Multisensory visual‐vestibular neurons have been shown to facilitate near‐optimal cue integration during self‐motion perception (monkeys: Angelaki et al., 2009). Within the vestibular nuclei, position vestibular pause (PVP) neurons receive direct ipsilateral vestibular inputs and project to contralateral motoneurons, which innervate extraocular muscles, thereby supporting the VOR (monkeys: Cullen & Roy, 2004; McCrea et al., 1987). A direct relationship between PVP interneuron sensitivity and VOR gain has been established (monkeys: Roy & Cullen, 1998). Interestingly, it has been shown that PVP neurons also discharge during OKN and optokinetic after‐nystagmus (OKAN) tests, indicating that neuronal mechanisms are shared between VOR and OKN (monkeys: Raphan et al., 1979; Waespe & Henn, 1977; Yakushin et al., 2017). PVP neurons decrease their firing during VORS (monkeys: Roy & Cullen, 1998), likely due to inhibition from the cerebellar flocculus and ventral paraflocculus (monkeys: Lisberger et al., 1994a, 1994b). The cerebellar flocculus plays a pivotal role in visuo‐vestibular adaptation by integrating current sensory inputs from vestibular and non‐vestibular input with prior experiences (rabbits: Nagao, 1990; monkeys: Stone & Lisberger, 1990; mice: Katoh et al., 1998; Shin et al., 2011). Based on this framework, our sensory conflict paradigm likely involves discrepancy detection within the cerebellar flocculus, which in turn directly modulates PVP neuron activity. Notably, the downregulation of oculomotor reflexes observed here is not generalized and does not suggest a broad reduction in PVP neuron sensitivity. Instead, it is specific to the source of conflict and the affected sensory modality. This idea is supported by studies demonstrating a 30% decrease in PVP neuron modulation during VORS tasks (involving both visual and vestibular inputs) compared to VOR in darkness (relying solely on vestibular inputs) (monkeys: Cullen & McCrea, 1993; Scudder & Fuchs, 1992). Following VR exposure, inhibition of PVP neurons – facilitated by a fixation point – would be reinforced in the VORS task, without altering PVP activity during the OKN task. Conversely, after vestibular challenge during PF, the activation of PVP neurons by visual field motion would be diminished in the OKN task, yet this reduction would not facilitate the downregulation of PVP firing in response to vestibular input during the VORS task.

Given the shared neural pathways and integrative mechanisms between VOR and OKN, it is essential to understand how individual sensory sensitivities influence the detection of discrepancies, multisensory integration processes, and ultimately, susceptibility to sensory conflicts. Our findings reveal a significant relationship between baseline OKN variables and cybersickness severity, with individuals reporting higher nausea scores exhibiting stronger OKN performance metrics. This suggests that heightened visual sensitivity may predispose individuals to more pronounced visuo‐vestibular conflicts, aligning with recent findings by Fulvio et al. (2021), who reported a positive correlation between visual cue sensitivity and cybersickness severity in virtual reality. However, in the parabolic flight setting, OKN variables are not significantly correlate with questionnaire score differences, indicating that visual dominance alone may not fully explain MS susceptibility in altered gravitational environments. In contrast, VORS performances, which are not reliable predictors of cybersickness severity, correlate with space‐sickness severity. During parabolic flight, lower baseline vertical eye instability (EDV) is strongly associated with a greater severity of nausea symptoms, suggesting that individuals with more effective VOR suppression may be more susceptible to SMS. Similarly, space motion sickness sensitivity was negatively correlated with both the latency of corrective saccades and the gain in the VORS task (data not shown), indicating a robust phenomenon. The reasons why participants with less robust VOR are also more sensitive to SMS remain unclear, but echo similar findings reported by Thornton and Uri (1991) where SMS susceptible individuals displayed higher corrective saccade frequency than non‐susceptible participants in a VORS task. Collectively, these studies suggest that visual and vestibular sensitivity play a crucial role in MS susceptibility, with the relevant sensory modality depending on the specific type of MS.

In conclusion, our study demonstrates that even relatively short exposure to a sensory conflict capable of inducing motion sickness is sufficient to reshape the integration of the involved sensory modalities. The post‐adaptive differences observed following terrestrial or space motion sickness support the idea of distinct sensory origins, despite similar symptomatology. These sensory specificities distinguishing space motion sickness from terrestrial motion sickness, along with the associated individual susceptibilities, are essential prerequisites for the development of more refined, targeted preventive treatments for each type of motion sickness, as well as for predicting individual sensitivity to novel situations such as space travel.

## AUTHOR CONTRIBUTIONS

Tess Bonnard and Etienne Guillaud: Conceived and designed the study, performed experiments, analyzed data, conducted statistical analyses, interpreted results and wrote manuscript. Emilie Doat: Performed experiments Jean‐René Cazalets: Contributed to results interpretation and assisted with manuscript revisions Dominique Guehl: Assisted in project conception and participant inclusion visit. All authors have read and approved the final version of this manuscript and agree to be accountable for all aspects of the work in ensuring that questions related to the accuracy or integrity of any part of the work are appropriately investigated and resolved. All persons designated as authors qualify for authorship, and all those who qualify for authorship are listed.

## CONFLICT OF INTEREST

None declared.

## Data Availability

The datasets analysed during the present study are available in the *Recherche Data Gouv* repository at the following address: https://doi.org/10.57745/QSX9C9.
